# Face-/Edge-Shared 3D Perovskitoid Single Crystals with Suppressed Ion Migration for Stable X-Ray Detector

**DOI:** 10.1007/s40820-025-01788-z

**Published:** 2025-06-23

**Authors:** Zimin Zhang, Xiaoli Wang, Huayang Li, Dong Li, Yang Zhang, Nan Shen, Xue-Feng Yu, Yucheng Liu, Shengzhong Liu, Haomin Song, Yanliang Liu, Xingzhu Wang, Shi Chen

**Affiliations:** 1 Henan Key Laboratory of Quantum Materials and Quantum Energy, School of Future Technology, Henan University, Zhengzhou, 450046 People’s Republic of China; 2 Shenzhen Institute of Advanced Technology, Chinese Academy of Sciences, Shenzhen, Guangdong 518055 People’s Republic of China; 3 University of Chinese Academy of Sciences, Beijing, 100049 People’s Republic of China; 4 Key Laboratory of Biomedical Imaging Science and System, Chinese Academy of Sciences, Shenzhen, Guangdong 518055 People’s Republic of China; 5 Key Laboratory of Applied Surface and Colloid Chemistry, Shaanxi Engineering Lab for Advanced Energy Technology, School of Materials Science and Engineering, National Ministry of Education, Shaanxi Normal University, Xi’an, 710119 People’s Republic of China; 6 State Key Laboratory of Catalysis, Dalian National Laboratory for Clean Energy, Dalian Institute of Chemical Physics, Chinese Academy of Sciences, Dalian, 116023 People’s Republic of China; 7 Institute of Applied Physics and Materials Engineering, University of Macau, Taipa, Macau, SAR 999078 People’s Republic of China; 8 School of Electrical Engineering, University of South China, Hengyang, 421001 People’s Republic of China

**Keywords:** 3D perovskitoid, Single crystals, Suppressed ion migration, High operating stability, X-ray detector

## Abstract

Although three-dimensional metal halide perovskites are promising candidates for direct X-ray detection, the ion migration of perovskites seriously affects the detector stability. Herein, face-/edge-shared 3D heterometallic glycinate hybrid perovskitoid Pb_2_CuGly_2_X_4_ (Gly = -O_2_C-CH_2_-NH_2_; X = Cl, Br) single crystals (SCs), in which the adjacent lead halide layers are linked by large-sized Cu(Gly)_2_ pillars, are synthesized in water. The Cu(Gly)_2_ pillars in combination with face-/edge-shared inorganic skeleton are found able to synergistically suppress the ion migration, delivering a high ion migration activation energy (*E*_*a*_) of 1.06 eV. The Pb_2_CuGly_2_Cl_4_ SC X-ray detector displays extremely low dark current drift of 1.20 × 10^–9^ nA mm^−1^ s^−1^ V^−1^ under high electric field (120 V mm^−1^) and continuous X-ray irradiation (2.86 Gy), and a high sensitivity of 9,250 μC Gy^−1^ cm^−2^ is also achieved. More excitingly, the Pb_2_CuGly_2_Cl_4_ nanocrystal can be easily dispersed in water and directly blade-coated on thin-film transistor (TFT) array substrate, and the obtained Pb_2_CuGly_2_Cl_4_-based TFT array detector offers an X-ray imaging capability with spatial resolution of 2.2 lp mm^−1^.

**Supplementary Information:**

The online version contains supplementary material available at 10.1007/s40820-025-01788-z.

## Highlights

- 3D perovskitoid Pb_2_CuGly_2_X_4_ (Gly = -O_2_C-CH_2_-NH_2_; X = Cl, Br) single crystals (SCs) with face-/edge-shared inorganic skeleton and CuGly_2_ pillars delivered a high ion migration activation energy of 1.06 eV.
- The Pb_2_CuGly_2_Cl_4_ SC X-ray detector presented low dark current drift (1.20 × 10^–9^ nA mm^−1^ s^−1^ V^−1^ at 120 V mm^−1^) and high sensitivity (9250 μC Gy^−1^ cm^−2^).
- The Pb_2_CuGly_2_Cl_4_ film-based thin-film transistor array detector offered an X-ray imaging capability with spatial resolution of 2.2 lp mm^−1^.

**Supplementary Information:**

The online version contains supplementary material available at 10.1007/s40820-025-01788-z.

## Graphical abstract

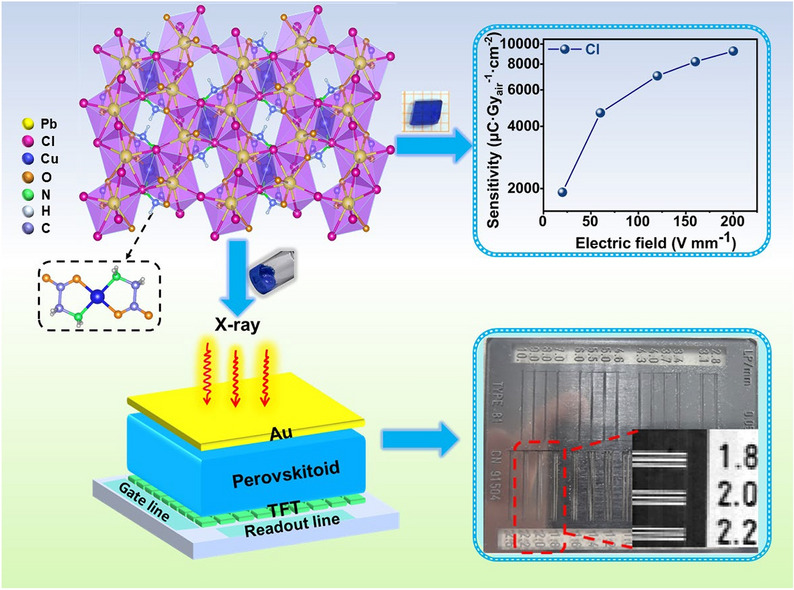

## Introduction

X-ray detection, as an efficient nondestructive technique, has been widely applied in various fields such as industrial inspection, medical diagnosis, security checks and homeland defense [1]. The lead halide perovskites, as emerging but efficient photovoltaic materials, have more potential in assembling next-generation cost-effective and highly sensitive X-ray detector due to their high X-ray attenuation coefficient, high defect tolerance, large carrier mobility–lifetime product (*μτ*), diverse dimensions and facile preparation process as well as inexpensive raw materials. Three-dimensional (3D) perovskite single crystals (SCs) including methylammonium lead bromide (MAPbBr_3_), methylammonium lead iodide (MAPbI_3_), cesium lead bromide (CsPbBr_3_) and formamidine lead iodide (FAPbI_3_) have been successfully synthesized to manufacture SC-based X-ray detectors [2–8], and for instance, the high sensitivity of 3D perovskite SC-based X-ray detector has been realized, displaying more than three orders of magnitude higher than that of commercial *α*-Se-based detector [9, 10]. However, the conventional 3D perovskite SCs are ionic compounds, and they are prone to ion migration under applied electric field [11]. This phenomenon will cause the baseline drift so as to the instability of response signals, which largely hampers the long-term operating stability of detectors, thereby limiting their commercial application [12].

Based on this situation, various strategies including device architecture optimization [10, 13], regulation of the quality of perovskite SCs [6, 8, 11, 14], interface passivation [7] and heterojunction design [9, 15, 16] have been proposed to inhibit the ion migration in perovskites, but the derived dark current drift of 3D perovskite SCs under large electric field still remains a daunting challenge. Therefore, the crystal structure design of perovskite materials is a fundamental method to sufficiently suppress intrinsic ion migration and construct stable X-ray detector.

One latest research work demonstrated that the inorganic networks in perovskites with edge- and face-sharing mode were more robust than that with corner-sharing mode, which is able to effectively impede the ion migration [17]. Besides, the large-sized organic cations in perovskites were also found able to efficiently block the ion migration [18–20]. For example, Zhang et al. introduced DGA (dimethylbiguanide) as an organic cation and fabricated the 2D perovskite SC (DGA)PbI_4_. The (DGA)PbI_4_ SC detector displayed a negligible dark current drift under an electric field of 200 V mm^−1^ for 5,426 s due to the suppressed ion migration [19]. However, this category of perovskite crystal structures combining edge-/face-sharing skeleton with large-sized cations is almost discovered in low-dimensional perovskites [21, 22]. Considering the low detection sensitivity of low-dimensional perovskites induced by the limited charge transport, constructing 3D perovskites with both edge-/face-sharing skeleton and large-sized cations to fully suppress the ion migration without sacrificing the sensitivity is highly imperative.

Since large-sized zwitterionic amino acids ligands have electron-rich amine and carboxylate groups, they can not only coordinate with the metal ion to form robust organic metal cross-linked cluster as a large-sized spacer [23–25], but also form strong bonding with lead halide polyhedrons and result in the formation of edge-/face-sharing skeleton [26], basically satisfying the above-mentioned structure design rule of 3D perovskites. Based on this idea, the 3D perovskitoid SC Pb_2_CuGly_2_Br_4_ (Gly = -O_2_C-CH_2_-NH_2_) was developed and reported to be a promising material for X-ray detection [27, 29]. However, the photoelectric properties of halide-modulated Pb_2_CuGly_2_X_4_ (X = Cl, Br) SCs and their final X-ray detection performance have not yet been systematically explored. Given these considerations, herein, we synthesized high-quality 3D heterometallic glycinate hybrid perovskitoid Pb_2_CuGly_2_X_4_ (X = Cl, Br) SCs via water evaporation method. The lead halide layers are linked by the robust Cu(Gly)_2_ units via the Pb–O bonds, leading to the formation of 3D framework. The Cu(Gly)_2_ pillars and robust inorganic networks enabled by face-/edge-shared [PbX_5_O_3_]^9−^ dodecahedron synergistically are able to effectively inhibit the ion migration, resulting in a high ion activation energy (*E*_*a*_). The Pb_2_CuGly_2_Cl_4_ SC exhibits lower microstrain, lower defect density, higher *E*_*a*_ and higher resistivity than Pb_2_CuGly_2_Br_4_ SC. As a result, the Pb_2_CuGly_2_Cl_4_ SC-based X-ray detector displays a high sensitivity of 9,250 μC Gy^−1^ cm^−2^. Meanwhile, the detector presents excellent operating stability under a high electric field (120 V mm^−1^) and continuous X-ray irradiation along with an extremely low dark current drift of 1.20 × 10^–9^ nA mm^−1^ s^−1^ V^−1^. In addition, an aqueous paste based on Pb_2_CuGly_2_Cl_4_ nanocrystal is prepared, and it is blade-coated on thin-film transistor (TFT) array substrate, achieving an X-ray imaging panel with spatial resolution of 2.2 lp mm^−1^.

## Experimental Section

### Materials

Lead chloride (PbCl_2_, 99%), copper bromide (CuBr_2_, 99%), anhydrous copper chloride (anhydrous CuCl_2_, 98%) and glycine (NH_2_CH_2_COOH, 99%) were purchased from Aladdin Reagent Ltd. Lead bromide (PbBr_2_, 99%) was purchased from Shanghai Maclin Biochemical Technology Co., LTD. All materials were used without further purification.

### Preparation of Pb_2_CuGly_2_Br_4_ and Pb_2_CuGly_2_Cl_4_ SCs

#### ***Preparations of the Pb***_***2***_***CuGly***_***2***_***Br***_***4***_*** SC***

0.3763 g lead bromide (PbBr_2_), 0.446 g copper bromide (CuBr_2_) and 0.3754 g glycine (NH_2_CH_2_COOH), with a molar ratio of 1:2:5, were dissolved in 90 mL distilled water and magnetically stirred at 100 °C for 30 min. The precursor solution was then rapidly evaporated at 100 °C for 24 h to form some micron-scale SCs. Thereafter, the supersaturated solution was rapidly filtered into a 20-mL vial and a small seed crystal was added to the supersaturated solution. After slow evaporation at 50 °C for 14 days, a large-sized Pb_2_CuGly_2_Br_4_ SC was obtained.

#### ***Preparations of the Pb***_***2***_***CuGly***_***2***_***Cl***_***4***_*** SC***

Similar to the preparation process of Pb_2_CuGly_2_Cl_4_ SC, 0.135 g anhydrous copper chloride (anhydrous CuCl_2_), 0.556 g lead chloride (PbCl_2_) and 0.7507 g glycine (NH_2_CH_2_COOH), with a molar ratio of 1:2:10, were dissolved in 60 mL distilled water and magnetically stirred at 100 °C for 30 min. The perovskite precursor solution was then rapidly evaporated at 100 °C for 24 h to form some micron-scale nucleus. Afterward, the supersaturated solution was rapidly filtered into a 20-mL vial and a small seed crystal was added to the solution. After slow evaporation at 50 °C for 14 days, a high-quality Pb_2_CuGly_2_Cl_4_ SC could be obtained.

### Device Fabrication

Firstly, the upper and bottom surfaces of the SCs was polished by using the grinding papers to remove the surface residue and defects. The thicknesses of the polished Pb_2_CuGly_2_Cl_4_ and Pb_2_CuGly_2_Br_4_ SCs were 1 and 0.8 mm, respectively. Then we fabricated the X-ray detectors with a Cu/SC/Cu vertical structure. The Cu electrodes with a thickness of about 60 nm were deposited on the both sides of the SCs by the thermal evaporation method, and the device area was controlled to be 1 mm^2^.

### Pb_2_CuGly_2_Cl_4_ TFT Array Detector Fabrication

Firstly, the Pb_2_CuGly_2_Cl_4_ SCs were grinded to powder. Then, the Pb_2_CuGly_2_Cl_4_ precursor paste with the concentration of 3.3 g mL^−1^ was obtained by directly dispersing Pb_2_CuGly_2_Cl_4_ in water. Finally, the Pb_2_CuGly_2_Cl_4_ paste was blade-coated on the TFT array followed by annealed 100 °C for 20 min to remove the residual water, and an Au electrode with thickness of 80 nm was deposited on Pb_2_CuGly_2_Cl_4_ film by thermal evaporation.

### Characterization of Materials

The single-crystal data of Pb_2_CuGly_2_X_4_ (X = Cl, Br) SCs were obtained from the previous literature [27]. The corresponding crystal structures of Pb_2_CuGly_2_X_4_ (X = Cl, Br) SCs were solved by the VESTA software. The powder X-ray diffraction (XRD) patterns of Pb_2_CuGly_2_X_4_ (X = Cl, Br) were performed by the D8-ADVANCE X-ray diffractometer with a Cu Kα radiation (λ = 1.54056 Å). The XRD data were collected at 293 K with a scan speed of 5° min^−1^ from 5° to 50°. The thermogravimetric analysis** (**TGA) was performed under a TA SDT650 instrument. 10 mg Pb_2_CuGly_2_X_4_ (X = Cl, Br) powder samples was placed in an alumina crucible followed by heated from 20 to 800 °C at a heating rate of 10 °C min^−1^ under an atmosphere of air. The UV–Vis–NIR absorbance spectra of Pb_2_CuGly_2_X_4_ (X = Cl, Br) crystal powder were obtained with a Cary 5000 UV–Vis–NIR spectrophotometer. The high-resolution ultraviolet photoelectron spectroscopy (UPS) was measured by an integrated vacuum system equipped with a multi-technique surface analysis system (VG ESCALAB MK II spectrometer).

### Detector Performance Measurement

The bulk resistivity of Pb_2_CuGly_2_X_4_ (X = Cl, Br) SC detector was obtained by fitting the dark I–V curves obtained via a semiconductor parameter analyzer (PDA SC-PRO 7). For X-ray characterization, an anode X-ray tube (NE-X) was utilized the source. The tube acceleration voltage of the X-ray source was fixed at 50 kV_p_, and the emitted X-ray dose rates were adjusted from 160 to 1620 μGy s^−1^ by controlling the operational current. The radiation dose rate was calibrated by using a commercial X-ray dosimeter from Germany. The both perovskitoid SC detectors were tested under different applied bias, and the response current of the detector was recorded via the PDA SC-PRO 7. As for the imaging test, a TFT array substrate (LZ-03AMR) was used to acquire the images of the target quickly.

## Results and Discussion

### Crystal Structure of Pb_2_CuGly_2_X_4_ (X = Cl, Br)

**Fig. 1:**
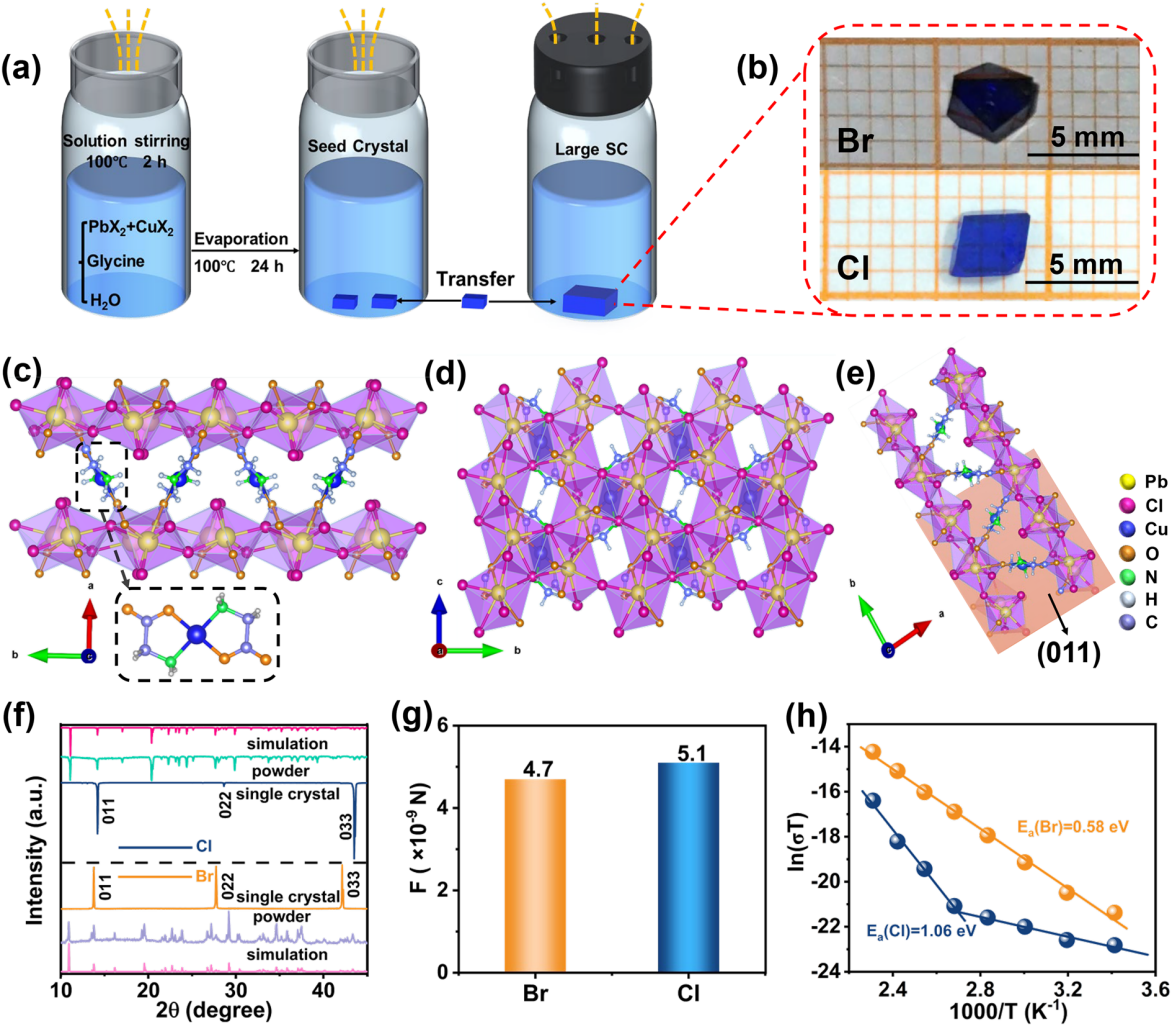
**a** Growth schematic of Pb_2_CuGly_2_X_4_ (X = Cl, Br). **b** Photographs of Pb_2_CuGly_2_X_4_ (X = Cl, Br) SCs. **c, d, e** Crystal structure of Pb_2_CuGly_2_Cl_4_ observed along c axis and a axis, respectively, and the (011) crystal plane. **f** XRD patterns of Pb_2_CuGly_2_X_4_ (X = Cl, Br) SC and powder. **g** Comparison of the electrostatic interaction force between Pb^2+^ and halide anions X^−^ (X = Cl, Br). **h** Temperature-dependent conductivity of Pb_2_CuGly_2_X_4_ (X = Cl, Br) SC devices

Figure 1a demonstrates the growth schematic of the perovskitoid SCs based on traditional solvent evaporation method according to a previous literature [27]. In brief, the PbX_2_, CuX_2_ (X = Cl, Br) and glycine were completely dissolved in water at 100 °C; then, the solution was rapidly volatilized for 24 h to form numerous seed crystals. Subsequently, the supersaturated solutions were filtered, and one seed crystal was transferred to the supersaturated solutions for further growth. After slow evaporation at 50 °C for 14 days, the large-sized perovskitoid SCs were obtained. Figure 1b gives the photographs of Pb_2_CuGly_2_Br_4_ SC and Pb_2_CuGly_2_Cl_4_ SC with the dimension sizes of about 4 mm × 3 mm × 1.2 mm and 3 mm × 2 mm × 0.7 mm, and the Pb_2_CuGly_2_Br_4_ SC exhibits dark blue, while the Pb_2_CuGly_2_Cl_4_ displays light blue. The Pb_2_CuGly_2_X_4_ (X = Cl, Br) crystallizes in the P2_1_/c group, and the detailed crystallographic information is presented in Table S1. As for the crystal structure of Pb_2_CuGly_2_Cl_4_, one copper ion (Cu^2+^) coordinated with two deprotonated Gly via amine groups and carboxylate groups to form robust CuGly_2_ symmetric unit, where the non-hydrogen atoms approximately are on a plane and the Cu^2+^ is located at the center of the unit. Then the lead chlorine layers are linked by the CuGly_2_ through Pb–O bonds to form the 3D frameworks (Fig. 1c), which is different from the traditional 3D perovskite structure. It should be noted that the lead atoms are surrounded by five chlorine atoms and three oxygen atoms to construct a [PbCl_5_O_3_]^9−^ dodecahedron (Fig. S2). Then these dodecahedrons build the inorganic layer via the face-sharing and edge-sharing connection (Fig. 1d). Except for the CuGly_2_ pillars, the robust inorganic networks enabled by face/edge-sharing also can significantly suppress the ion migration [17], which is conductive to inhibiting the dark current drift of the device. Figure 1e exhibits the (011) plane of the Pb_2_CuGly_2_Cl_4_ SC. The crystal structure of Pb_2_CuGly_2_Br_4_ is similar to Pb_2_CuGly_2_Cl_4_, and the detail structure of Pb_2_CuGly_2_Br_4_ is listed in the Fig. S1.

The XRD patterns of Pb_2_CuGly_2_X_4_ (X = Cl, Br) powder and SCs are well matched with their simulated ones obtained from single-crystal structure data [27]. In addition, the Pb_2_CuGly_2_X_4_ SCs show three strong diffraction peaks (0ll) (l = 1, 2, 3), implying their good crystallographic orientation along the (0ll) plane (Fig. 1f). It is worth noting that the (0ll) plane diffraction peaks of Pb_2_CuGly_2_Cl_4_ SC shift to the larger angles compared with Pb_2_CuGly_2_Br_4_ SC, conforming a smaller lattice constant of Pb_2_CuGly_2_Cl_4_ SC. The microstrain (*ε*) of Pb_2_CuGly_2_X_4_ SCs was calculated according to the XRD patterns by the Williamson–Hall equation [29]:

1 $$\beta {\text{cos}}\theta = k\lambda /D + { 4}\varepsilon {\text{sin}}\theta$$

where *β*, *θ*, *k*, *λ* and *D* represent the full width at half maximum (FWHM), Bragg’s diffraction angle, Scherrer constant, the wavelength of the X-ray and average crystallite size, respectively. The* ε* can be derived from the slope of the fitting line (Fig. S3). The Pb_2_CuGly_2_Cl_4_ exhibits smaller *ε* of 0.02 than Pb_2_CuGly_2_Br_4_ (0.07), indicating better crystalline quality of Pb_2_CuGly_2_Cl_4_ SC with lower crystal defect density. Subsequently, the thermogravimetric analysis (TGA) of Pb_2_CuGly_2_X_4_ (X = Cl, Br) powder was performed in Fig. S4. Pb_2_CuGly_2_Cl_4_ begins to decompose at 198 °C, exhibiting a similar decomposition temperature with that of Pb_2_CuGly_2_Br_4_ (194 °C). This is can be ascribed to the melting of the CuGly_2_ clusters [27].

The ideal X-ray detection materials should possess suppressed ion migration under large applied bias to maintain the stable output of the photocurrent. To assess the ion migration in the Pb_2_CuGly_2_X_4_ SCs, the individual electrostatic interaction force between the lead cations and halide ions was calculated according to the Coulomb’s law. The corresponding ionic radii and valence states are listed in Table S2, and Fig. S5 exhibits the schematic diagram of the electrostatic interaction force. As shown in Fig. 1g, the Cl^−^ has a stronger interaction with Pb^2+^ (5.1 × 10^–9^ N) than the Br^−^ (4.7 × 10^–9^ N) owing to its smaller ionic radii, which may results in inhibited ion migration so as to enhanced operating stability for Pb_2_CuGly_2_Cl_4_ SC detector. Furthermore, we conducted the temperature-dependent conductivity measurement to compare the ion migration activation energy (*E*_*a*_) of the two SCs (Fig. 1h). The *E*_*a*_ can be calculated according to the Nernst–Einstein equation [24]:

2 $$\sigma \left(T\right)=\frac{{\sigma }_{0}}{T}\text{exp}\left(\frac{-{E}_{a}}{{k}_{B}T}\right)$$

where the σ represents the measured conductivity of the SCs under different temperature, σ_0_ is a constant and the *k*_*B*_ reflects the Boltzmann constant. The calculated *E*_*a*_ of Pb_2_CuGly_2_Cl_4_ and Pb_2_CuGly_2_Br_4_ SC are 1.06 eV and 0.58 eV, respectively, which is in accord with the results concluded from Fig. 1f, g. The higher *E*_*a*_ of Pb_2_CuGly_2_Cl_4_ allows the utilization of large bias to drive the carrier transport without sacrificing the detector operating stability.

### Optoelectronic Properties of Pb_2_CuGly_2_X_4_ (X = Cl, Br)

**Fig. 2:**
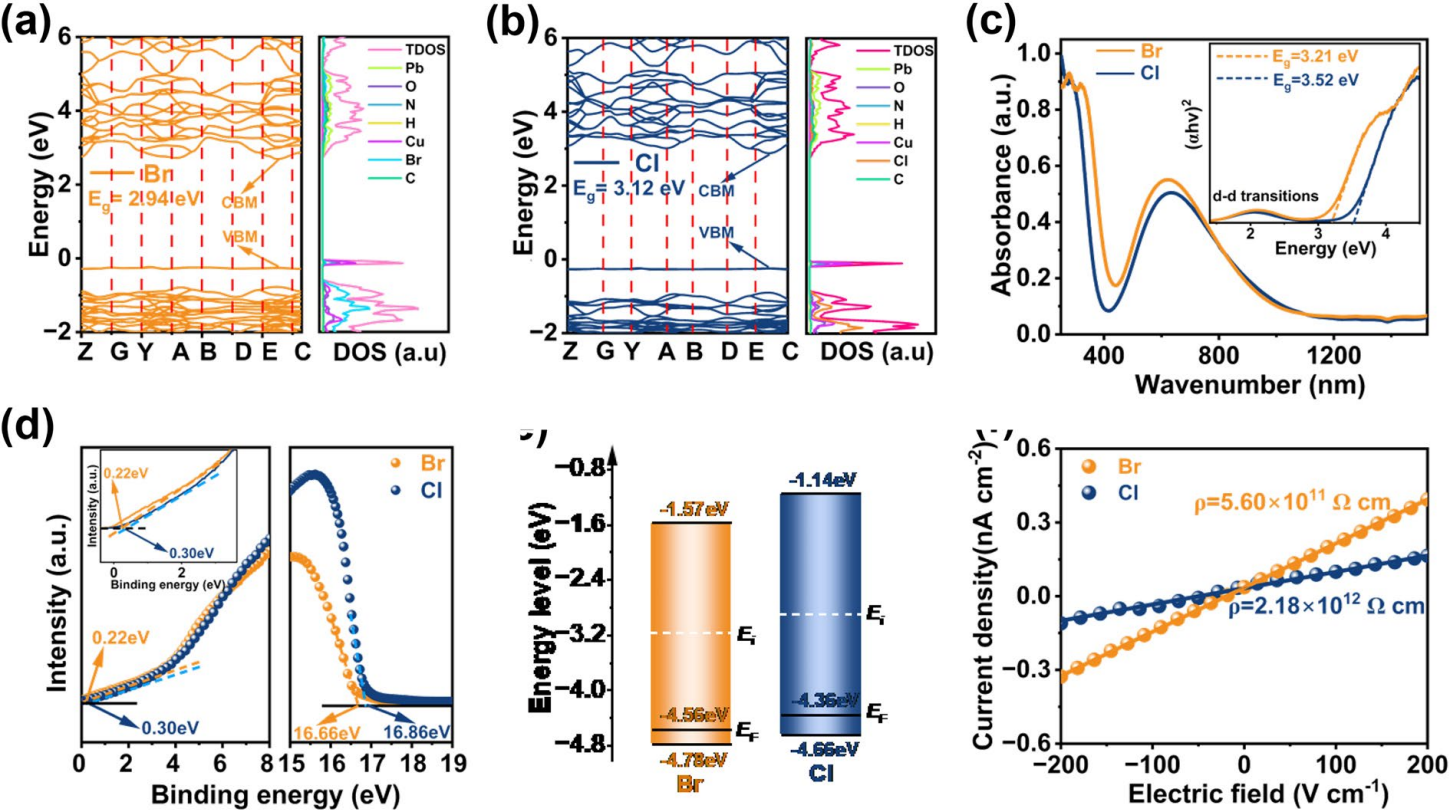
**a** Band structure and DOS simulation of Pb_2_CuGly_2_Br_4_. **b** Band structure and DOS simulation of Pb_2_CuGly_2_Cl_4_. **c** UV–Vis–NIR absorbance spectra of Pb_2_CuGly_2_X_4_ (X = Cl, Br) single-crystal powder, and the inset is the corresponding Taus plots. **d** UV photoelectron spectroscopy of Pb_2_CuGly_2_X_4_ (X = Cl, Br) and the partial enlarged detail (inset). **e** Energy level diagram of Pb_2_CuGly_2_X_4_ (X = Cl, Br). **f** Dark current density as a function of the electric field for Pb_2_CuGly_2_X_4_ (X = Cl, Br) SC X-ray detectors

The band structure of Pb_2_CuGly_2_X_4_ (X = Cl, Br) was explored by density functional theory (DFT) calculations, and the detailed calculated method is presented in supporting information. As shown in Fig. 2a, b, the top valence band is a flat band for Pb_2_CuGly_2_X_4_ (X = Cl, Br), and both Pb_2_CuGly_2_Br_4_ and Pb_2_CuGly_2_Cl_4_ are direct semiconductors with a calculated band gap of 2.94 and 3.12 eV, respectively. Such band gaps are consistent with the blue color of the crystals. It should be noted that the calculated band gap of Pb_2_CuGly_2_Br_4_ in this work is relative lower than that (3.37 eV) reported in reference [29] along with the difference in their electronic band structures [29], which may be attributed to the different level theory employed. According to the calculated density of states (DOS) spectra of Pb_2_CuGly_2_X_4_ (X = Cl, Br), the Pb 5*d* orbital mainly accounts for the conduction band minimum (CBM) of Pb_2_CuGly_2_X_4_ (X = Cl, Br), whereas the Cu 3*d* and O 2*p* orbitals mainly dominate the corresponding valence band maximum (VBM). This clearly declares that CuGly_2_ species can participate the carrier’s transport. Afterward, we measured the optical *E*_*g*_ of Pb_2_CuGly_2_X_4_ (X = Cl, Br) by the ultraviolet–visible–near infrared (UV–Vis–NIR) absorbance spectrum (Fig. 2c). It can be seen that Pb_2_CuGly_2_X_4_ (X = Cl, Br) exhibits a broad absorption range from visible to near infrared region, where the high energy absorption originates from the matrix absorption, and the low energy absorption is due to the *d–d* transitions of Cu^2+^ [29]. The blue color of the Pb_2_CuGly_2_X_4_ (X = Cl, Br) SC can be attributed to the d–d transitions of Cu^2+^ [27, 28]. Based on the matrix absorption, the optical *E*_*g*_ of Pb_2_CuGly_2_X_4_ (X = Cl, Br) is calculated to be 3.52 and 3.21 eV, respectively. The actual value of the band gap is always higher than that derived from DFT calculation, which has been widely accepted owing to the limitation of the employed model. The calculated *E*_*g*_ of Pb_2_CuGly_2_Br_4_ is in accord with that (3.25 eV) in reference [29]. The luminescent properties of Pb_2_CuGly_2_X_4_ (X = Cl, Br) SC are also investigated. As shown in Fig. S6, the Pb_2_CuGly_2_X_4_ (X = Cl, Br) SC under the illumination of 310 nm ultraviolet (UV) light exhibits no fluorescence except for their intrinsic blue color, and the fluorescence emission peak of Pb_2_CuGly_2_X_4_ (X = Cl, Br) single crystals is absent in the range of 320–600 nm, 640–850 nm under the excitation of 310 nm UV light, indicating no fluorescence emission. Then the UV photoelectron spectroscopy (UPS) measurements were taken to further estimate the energy level of Pb_2_CuGly_2_X_4_ (X = Cl, Br) (Fig. 2d). It illustrates that the work function of Pb_2_CuGly_2_X_4_ (X = Cl, Br) is −4.36 and −4.56 eV, respectively, from the secondary electron cutoff edge of the spectrum, and the valence band of Pb_2_CuGly_2_X_4_ (X = Cl, Br) is 0.3 and 0.22 eV below the Fermi level, respectively. Based on the results of UPS spectrum and absorption spectra, the VBM and CBM of Pb_2_CuGly_2_X_4_ (X = Cl, Br) are located at −4.66/−4.78 eV and −1.14/−1.57 eV, respectively (Fig. 2e). Since the Fermi levels of Pb_2_CuGly_2_X_4_ (X = Cl, Br) are lower than the middle positon of forbidden band, that indicates the Pb_2_CuGly_2_X_4_ (X = Cl, Br) are p-type semiconductors. Subsequently, the resistivity of Pb_2_CuGly_2_X_4_ (X = Cl, Br) SC was characterized. As shown in Fig. 2f, the resistivity of Pb_2_CuGly_2_X_4_ (X = Cl, Br) is derived to be 2.18 × 10^12^ and 5.6 × 10^11^ Ω cm, respectively, which is much higher than that of 3D perovskites like MAPbI_3_, MAPbBr_3,_ FAPbI_3_, etc. [31, 32]. The higher resistivity of Pb_2_CuGly_2_Cl_4_ can be assigned to the higher crystal quality and suppressed ion migration, which is essential to reduce the detector noise.

### Performance of Pb_2_CuGly_2_X_4_ SC X-ray Detector

**Fig. 3:**
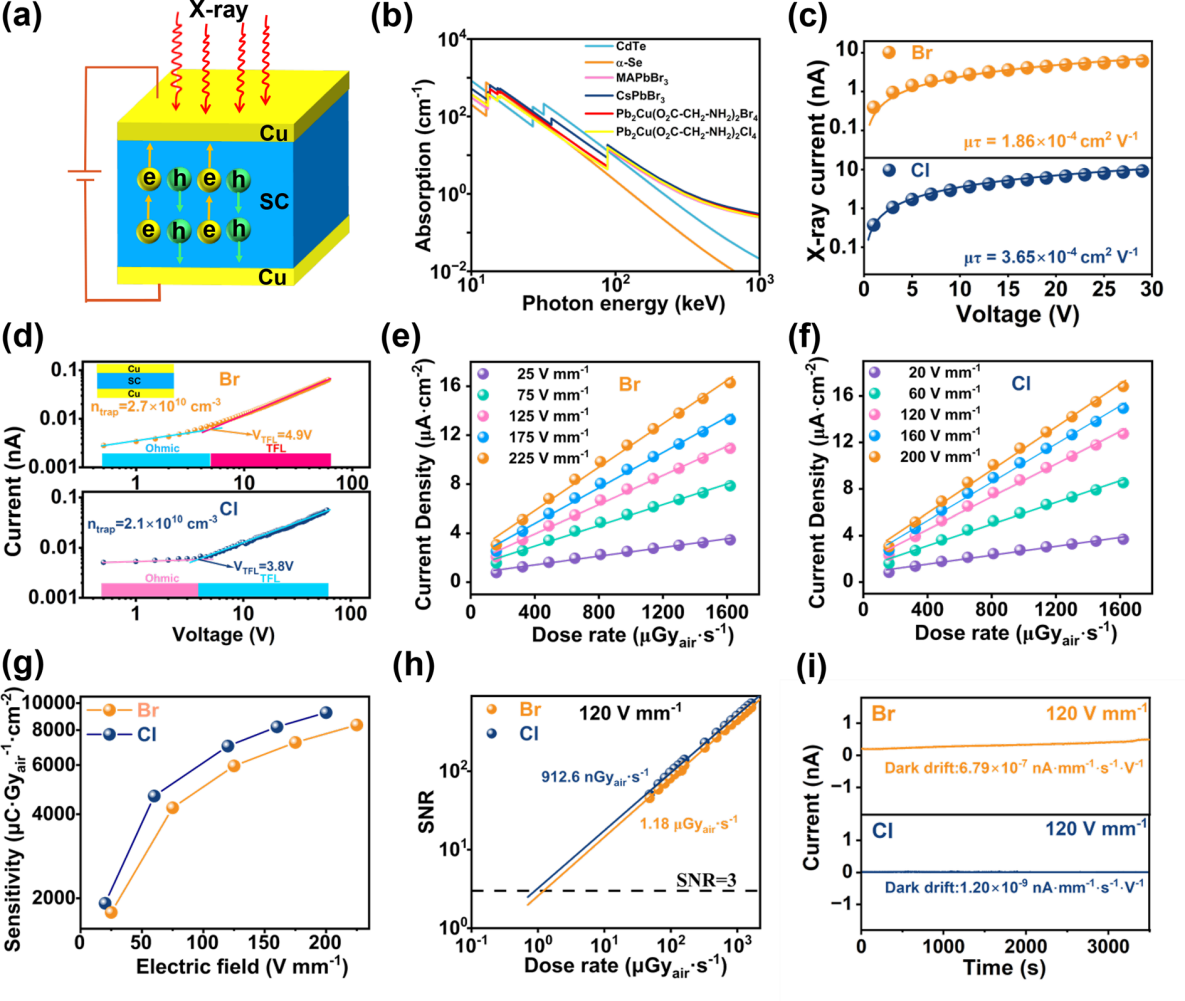
**a** Schematic of detector structure and working mechanism. **b** Absorption coefficient of CdTe, α-Se, Pb_2_CuGly_2_X_4_ (X = Cl, Br), MAPbBr_3_ and CsPbBr_3_. **c** *μτ* product measurement of Pb_2_CuGly_2_X_4_ (X = Cl, Br) SC. **d** Dark current–voltage characteristics of Pb_2_CuGly_2_X_4_ (X = Cl, Br) SC detector measured by the SCLC method, inset is the device structure. **e** Photocurrent density of Pb_2_CuGly_2_Br_4_ SC detector under various dose rates. **f** Photocurrent density of Pb_2_CuGly_2_Cl_4_ SC detector under various dose rates. **g** Sensitivity of Pb_2_CuGly_2_X_4_ (X = Cl, Br) SC detector under different electric fields. **h** SNR of the Pb_2_CuGly_2_X_4_ (X = Cl, Br) SC detector at different dose rates with a fixed electric field of 120 V mm^−1^. **i** Dark current stability measurement of Pb_2_CuGly_2_X_4_ (X = Cl, Br) SC detector at the electric field of 120 V mm.^−1^

We constructed the X-ray detector with device configuration of Cu/Pb_2_CuGly_2_X_4_ (X = Cl, Br) SC/Cu to estimate their detection performance. The working mechanism of the detector is rendered in Fig. 3a. When the detector absorbs the X-ray photons, numerous electron–hole pairs are generated, then these electron–hole pairs separate and transport across the semiconductors under the applied bias, and finally be collected by the electrodes. Figure 3b compares the X-ray attenuation coefficients of Pb_2_CuGly_2_X_4_ (X = Cl, Br) SC with other semiconductors like CdTe, α-Se, MAPbBr_3_ and CsPbBr_3_ over a broad photon energy region ranging from 10 to 10^3^ keV by using the NIST database [33]. When the X-ray photon energy ranges from 100 eV to 1000 keV, the X-ray absorption coefficients of Pb_2_CuGly_2_X_4_ (X = Cl, Br) are higher than that of CdTe and α-Se, and comparable to MAPbBr_3_ and CsPbBr_3_, exhibiting a strong X-ray stopping power. The high sensitivity of X-ray detectors depends on the efficient charge collection. To character the important physical property, the *μτ* products of the Pb_2_CuGly_2_X_4_ (X = Cl, Br) SC detector, reflecting the average carrier drift distance per unit electric field, were derived by fitting the photoconductivity of Pb_2_CuGly_2_X_4_ (X = Cl, Br) SC detector under the X-ray illumination and applied bias according to the modified Hecht equation [34]:

3 $$\text{I}=\frac{{I}_{0}\mu \tau V}{{L}^{2}}\frac{1-\text{exp}\left(-\frac{{L}^{2}}{\mu \tau V}\right)}{1+\frac{Ls}{V\mu }}$$

where the *I*_*0*_ denotes the saturated photocurrent, *V* is the applied bias, L represents the thickness of the Pb_2_CuGly_2_X_4_ (X = Cl, Br) SC and the *s* is the surface recombination velocity. The *μτ* product of Pb_2_CuGly_2_Cl_4_ SC detector is determined to be 3.65 × 10^–4^ cm^2^ V^−1^, which is higher than that (1.86 × 10^–4^ cm^2^ V^−1^) of the Pb_2_CuGly_2_Br_4_ SC detector (Fig. 3c). Therefore, the higher *μτ* product of Pb_2_CuGly_2_Cl_4_ SC device will result in a higher X-ray detection performance. Then, the relative dielectric constants (*ε*) of Pb_2_CuGly_2_X_4_ (X = Cl, Br) SC were assessed from the capacitance–frequency curves, as shown in Fig. S7. The *ε* values of Pb_2_CuGly_2_X_4_ (X = Cl, Br) SC are calculated to be 32 and 24, respectively. According to the Wannier–Mott exciton equation [35], the exciton binding energy (*E*_*b*_) is inversely proportional of the *ε*^*2*^. This demonstrates that Pb_2_CuGly_2_Cl_4_ may possess lower *E*_*b*_ compared to Pb_2_CuGly_2_Br_4_, facilitating the separation of electron–hole pairs. As discussed above, the Pb_2_CuGly_2_Cl_4_ SC shows smaller microstrain than Pb_2_CuGly_2_Br_4_ SC, implying lower trap density. To further confirm the speculation, we estimated the trap density (*n*_*trap*_) of Pb_2_CuGly_2_X_4_ (X = Cl, Br) SC by a space-charge-limited currents (SCLC) measurements in Fig. 3d. The Pb_2_CuGly_2_Cl_4_ SC presents lower *n*_*trap*_ (2.1 × 10^10^ cm^−3^) than that of Pb_2_CuGly_2_Br_4_ SC (2.7 × 10^10^ cm^−3^). The lower *n*_*trap*_ in Pb_2_CuGly_2_Cl_4_ SC is beneficial to restrain carrier recombination, thereby contributing to enhanced charge collection efficiency. In addition, the as-prepared Pb_2_CuGly_2_Br_4_ SC gives lower trap density in comparison with that (7.55 × 10^10^ cm^−3^) in the literature [29], indicating the improved crystal quality of Pb_2_CuGly_2_Br_4_ SC in this work.

The Pb_2_CuGly_2_X_4_ (X = Cl, Br) SC detectors exhibit an ideal linear photocurrent response with the varied X-ray dose rates from 160 to 1620 μGy s^−1^ under different electric field as in Figs. S8 and S9, and the sensitivity of Pb_2_CuGly_2_Cl_4_ SC detector is determined to be 1920, 4650, 7020, 8220 and 9250 μC Gy^−1^ cm^−2^ at the electric fields of 20, 60, 120, 160 and 200 V mm^−1^, respectively, according to the quantitative liner regression analyses (Fig. 3f). As a contrast, the Pb_2_CuGly_2_Br_4_ SC detector delivers lower sensitivities of 1780, 4220, 5960, 7230 and 8350 μC Gy^−1^ cm^−2^ under electric fields of 25, 75, 125, 175 and 225 V mm^−1^ than PbCuGly_2_Cl_4_ SC detector in Fig. 3e. It is worth noting that the sensitivity of our Pb_2_CuGly_2_Br_4_ SC detector is higher than that (1462.7 μC Gy^−1^ cm^−2^, 50 V mm^−1^) reported in the literature [29], which is consistent with the result of Fig. 3d. Furthermore, we compared the detection efficiency of the both detectors. The theoretical sensitivity (*S*_*0*_) of Pb_2_CuGly_2_X_4_ (X = Cl, Br) SC detector for 50 keV X-ray is estimated to be 4.85 × 10^3^ and 4.82 × 10^3^ μC Gy^−1^ cm^−2^, respectively. The detailed calculation information is provided in the experimental section. Thereby, the detection efficiency of Pb_2_CuGly_2_Cl_4_ SC detector under 120 V mm^−1^ is calculated to be 145% by *S*/*S*_*0*_, higher than that of Pb_2_CuGly_2_Br_4_ SC detector (~ 124%). It should be pointed out that both detectors present a trend that the sensitivity increases with the increase in the electric field due to the enhanced carrier collection efficiency (Fig. 3g). Thereby, higher sensitivity are supposed to be achieved once further increasing the electric field. We further compare the sensitivity of Pb_2_CuGly_2_Cl_4_ SC detector with other semiconductors like α-Se, 2D and 3D perovskite SCs (Table S3). The sensitivity of Pb_2_CuGly_2_Cl_4_ SC detector at 200 V mm^−1^ is over 21 times higher than that of commercial α-Se at 15,000 V mm^−1^ (440 μC Gy^−1^ cm^−2^) [36], demonstrating a promising application prospect. Although the sensitivity of Pb_2_CuGly_2_Cl_4_ SC detector is much larger than that of the reported 2D perovskite SCs such as (DGA)PbI_4_ (4,869 μC Gy^−1^ cm^−2^ @ 1,200 V mm^−1^) and (4AEPy)PbI_4_ (5,627μC Gy^−1^ cm^−2^@ 200 V mm^−1^) [19, 20], it is still behind the traditional 3D perovskite SCs like CsPbBr_3_ and FAPbI_3_ [3, 11], which is caused by the unique crystal structure of Pb_2_CuGly_2_Cl_4_ SC.

The low detection limit of the detector is a primary parameter of high-performance detector that determines the lowest detectable X-ray dose rates. According to the International Union of Pure and Applied Chemistry, the dose rate can be viewed the lowest detection limit when the signal-to-noise ratios (SNR) is 3 [37]. Therefore, we calculate the SNR of the Pb_2_CuGly_2_X_4_ (X = Cl, Br) SC detector at different dose rates with a fixed electric field of 120 V mm^−1^ followed by extending the fitting line to SNR = 3 (Fig. 3h). The detailed current density data used for calculating the detection limit of Pb_2_CuGly_2_X_4_ (X = Cl, Br) SC detector are provided in Fig. S10. The derived lowest detectable dose rate of Pb_2_CuGly_2_Cl_4_ SC detector is 912.6 nGy s^−1^, lower than that of Pb_2_CuGly_2_Br_4_ SC detector (1.18 μGy s^−1^), implying the excellent detection ability of Pb_2_CuGly_2_Cl_4_ SC at lower dose rate. The relative high detection limit of Pb_2_CuGly_2_X_4_ (X = Cl, Br) SC detectors can be ascribed to the defect-induced unstable time-resolved current density.

The large electric field always causes serious dark current drift of detector due to the well-known ion migration in perovskites. To inspect the dark current stability of Pb_2_CuGly_2_X_4_ (X = Cl, Br) SC detector, we applied an electric field of 120 V mm^−1^ on both detectors for 3,500 s. As shown in Fig. 3i, Pb_2_CuGly_2_Cl_4_ SC device shows lower dark current under 120 V mm^−1^ due to its larger bulk resistivity. In addition, the Pb_2_CuGly_2_Cl_4_ SC detector gives a lower dark current drift (*I*_*drift*_) of 1.20 × 10^–9^ nA mm^−1^ s^−1^ V^−1^ than that of Pb_2_CuGly_2_Br_4_ SC detector (6.79 × 10^–7^ nA mm^−1^ s^−1^ V^−1^), demonstrating higher dark current stability. The low *I*_*drift*_ of Pb_2_CuGly_2_Cl_4_ SC originates from stronger electrostatic interaction force, higher *E*_*a*_ and lower n_trap_, which can effectively suppress the dark current noise.

**Fig. 4:**
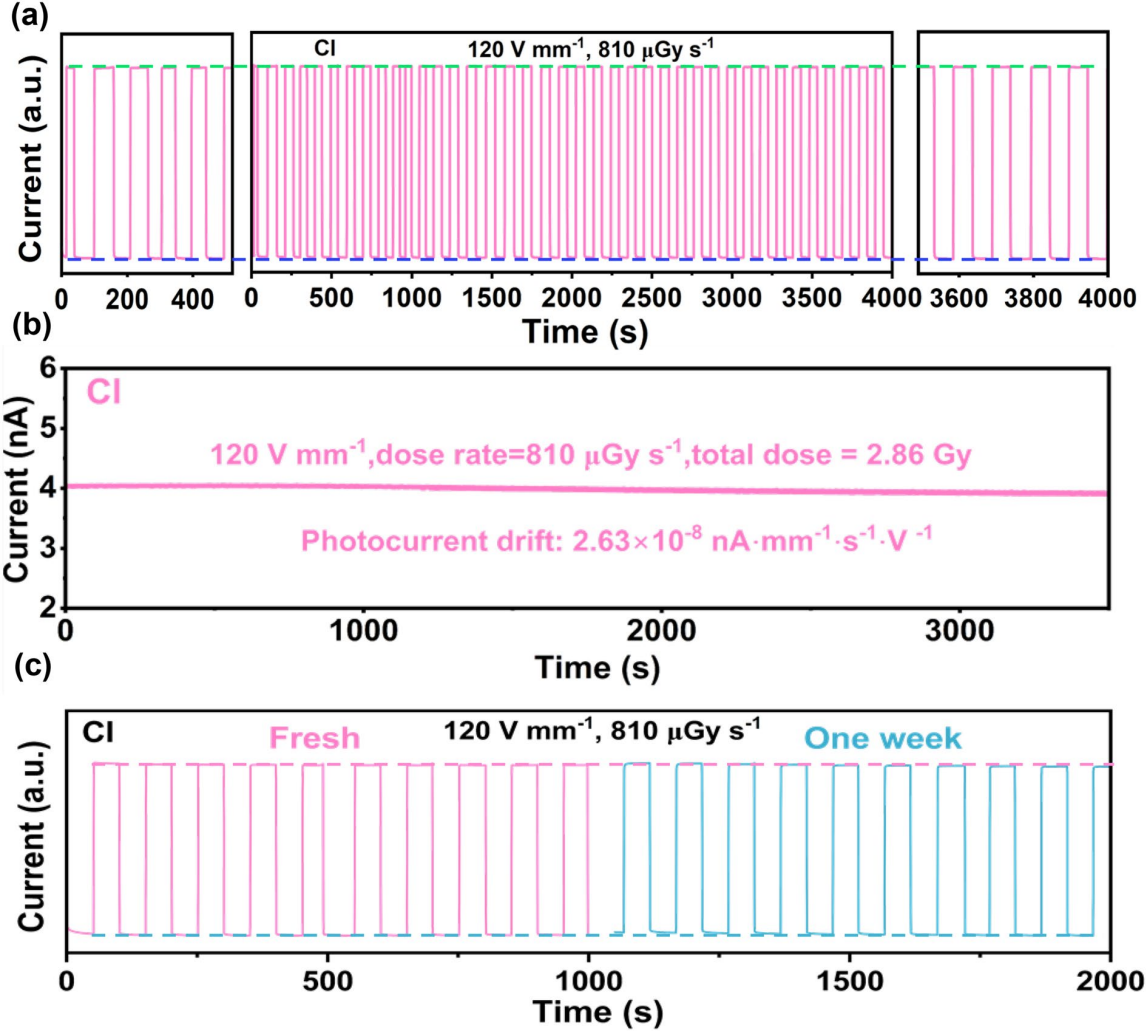
Response current of Pb_2_CuGly_2_Cl_4_ SC detector **a** under a pulsed X-ray irradiation at the electric field of 120 V mm^−1^ and **b** under continuous X-ray irradiation at the electric field of 120 V mm^−1^. **c** Air stability test of Pb_2_CuGly_2_Cl_4_ SC detector

The photocurrent response stability of the X-ray detector under pulsed X-ray irradiation is also a concern that should be cared. As illustrated in Fig. 4a, the Pb_2_CuGly_2_Cl_4_ SC detector displays stable photocurrent and dark current response with nearly no change during the whole measurement period of 4,000 s under the electric field of 120 V mm^−1^, suggesting an unprecedented X-ray response stability. In addition, we further tested the operating stability of the Pb_2_CuGly_2_Cl_4_ SC detector under continuous X-ray irradiation (810 μGy s^−1^) with the electric field of 120 V mm^−1^. As shown in Fig. 4b, the response current of Pb_2_CuGly_2_Cl_4_ SC detector almost maintains the initial photocurrent value after continuous work for over 3,500 s along with exposure to a total X-ray does of 2.86 Gy, which is equal to about 2.86 × 10^4^ exposure times of the standard commercial X-ray chest radiograph (~ 0.1 mGy per exposure) [38]. Similar to the estimation of dark current drift, the Pb_2_CuGly_2_Cl_4_ SC detector exhibits a photocurrent drift of 2.63 × 10^–8^ nA mm^−1^ s^−1^ V^−1^, indicating an excellent X-ray operating stability of the detector under the harsh working conditions. Finally, the long-term air stability test of Pb_2_CuGly_2_Cl_4_ SC detector was carried out. As shown Fig. 4c, the Pb_2_CuGly_2_Cl_4_ SC detector stored in air for one week without any encapsulation still exhibits no signal attenuation under the dose rate of 810 μGy s^−1^ at 120 V mm^−1^, demonstrating the excellent air stability of Pb_2_CuGly_2_Cl_4_ SC detector. Based on these excellent performances, the Pb_2_CuGly_2_Cl_4_ SC detector is expected to be suitable for high-quality X-ray imaging.

### X-ray Imaging of Pb_2_CuGly_2_Cl_4_ TFT Array Detector

**Fig. 5:**
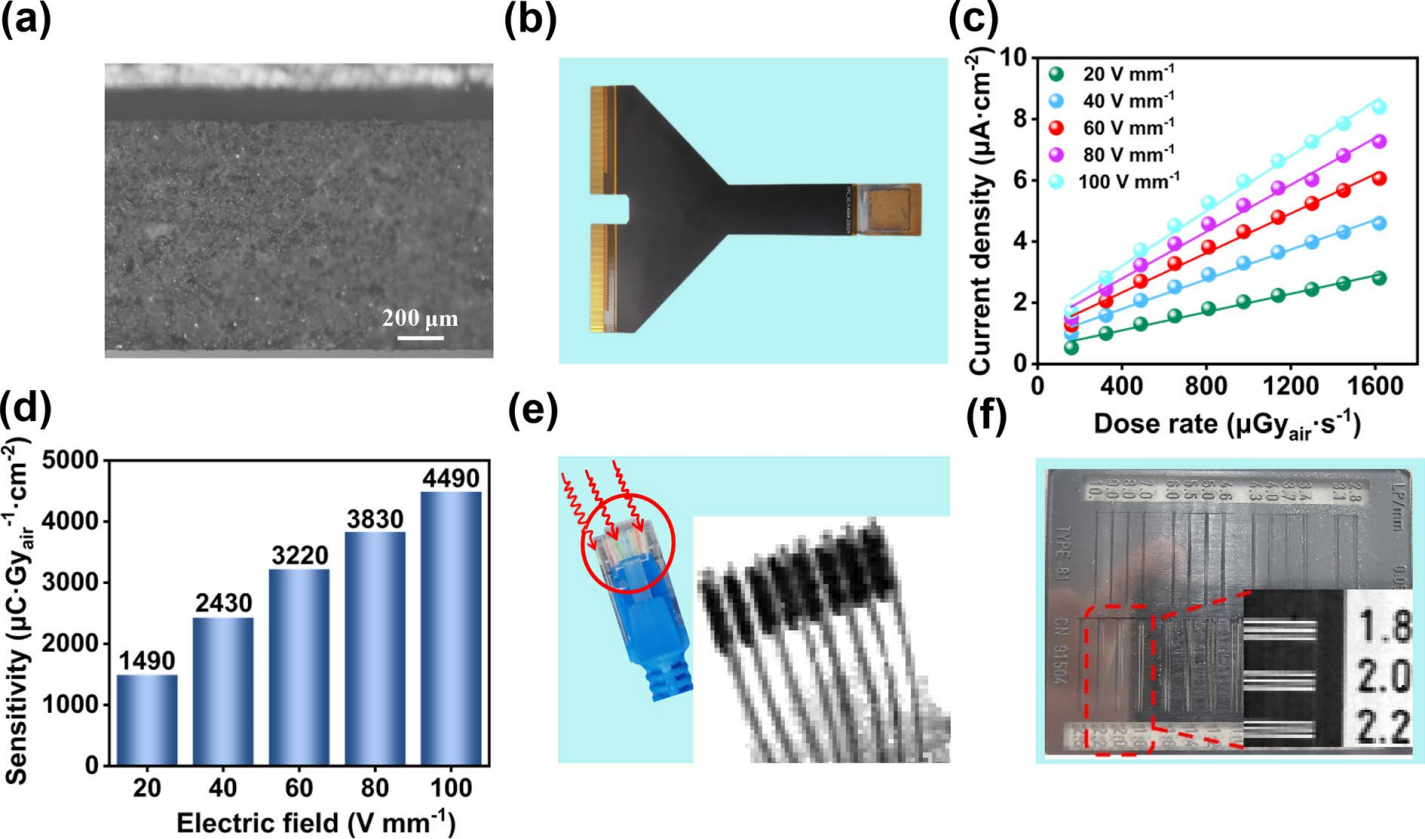
**a** Cross-sectional SEM of Pb_2_CuGly_2_Cl_4_ film. **b** Photograph of the Pb_2_CuGly_2_Cl_4_ TFT array detector. **c** Photocurrent density of Pb_2_CuGly_2_Cl_4_ TFT array detector under various dose rates at different electric field. **d** Sensitivity of Pb_2_CuGly_2_Cl_4_ TFT array detector under different electric field. **e** Optical photograph of the network cable plug and the corresponding X-ray image. **f** X-ray image of the lead line pair card

To meet the X-ray imaging practical application, developing integrated X-ray array detector is imperative. Considering the crystallization of Pb_2_CuGly_2_Cl_4_ SC in water, we prepared the Pb_2_CuGly_2_Cl_4_ aqueous paste by dispersing Pb_2_CuGly_2_Cl_4_ nanocrystal in water (Fig. S11). The Pb_2_CuGly_2_Cl_4_ paste was directly blade-coated onto the TFT array with pixel size of 200 × 200 μm^2^ (64 × 64 pixels), and the Pb_2_CuGly_2_Cl_4_ precursor film was heated at 100 °C to remove the residual water. Finally, a gold electrode was evaporated on the Pb_2_CuGly_2_Cl_4_ thick film (Fig. S12). As shown in Fig. 5a, the thickness of the Pb_2_CuGly_2_Cl_4_ thick film was about 1 mm. The XRD patterns of Pb_2_CuGly_2_Cl_4_ film is provided in the Fig. S13. It is discovered that the diffraction peak of Pb_2_CuGly_2_Cl_4_ film is consistent with that of the Pb_2_CuGly_2_Cl_4_ powder and the simulation, indicating no new phases or substances are formed during the film preparation process. This also confirms the excellent stability of Pb_2_CuGly_2_Cl_4_ powder in water. Figure 5b displays the photograph of the Pb_2_CuGly_2_Cl_4_ TFT array detector, and the microphotograph of the different region of TFT array is given in Fig. S14. Firstly, the X-ray detection performance of Pb_2_CuGly_2_Cl_4_ TFT array detector was studied. As shown in Fig. S15, the Pb_2_CuGly_2_Cl_4_ film TFT detector also exhibited an ideal linear photocurrent response with the varied X-ray dose rate under different applied bias, yielding a sensitivity of 1490, 2430, 3220, 3830 and 4490 μC Gy^−1^ cm^−2^ at the electric field of 20, 40, 60, 80 and 100 V mm^−1^(Fig. 5c, d). Compared with the Pb_2_CuGly_2_Cl_4_ SC detector, the Pb_2_CuGly_2_Cl_4_ TFT array detector gives lower sensitivity, which can be owing to the defects in Pb_2_CuGly_2_Cl_4_ polycrystalline film. Therefore, further optimizing the quality of Pb_2_CuGly_2_Cl_4_ film is expected to improve the sensitivity of Pb_2_CuGly_2_Cl_4_ TFT array detector.

Subsequently, we further performed the X-ray imaging study. A network cable plug is selected as the target for X-ray imaging, and the internal circuit of the network cable plug can be observed clearly by the X-ray image (Fig. 5e). To further estimate the spatial resolution of the Pb_2_CuGly_2_Cl_4_ TFT array detector, the lead line pair card is utilized as the X-ray imaging target, and its maximum spatial resolution is up to 2.2 lp mm^−1^ in Fig. 5f. It should be noted that the spatial resolution is largely related to the pixel size of the TFT array, and further decreasing the pixel size can achieve a better spatial resolution.

## Conclusions

In conclusion, we successfully synthesized high-quality 3D perovskitoid SCs Pb_2_CuGly_2_X_4_ (Gly = -O_2_C-CH_2_-NH_2_; X = Cl, Br) through amino acids ligands metal cross-linked strategy. The rigid Cu(Gly)_2_ pillars and robust inorganic networks enabled by the face/edge-shared [PbX_5_O_3_]^9−^ dodecahedron can synergistically inhibit the ion migration, leading to a high *E*_*a*_ of 1.06 eV. Compared with Pb_2_CuGly_2_Br_4_, the Pb_2_CuGly_2_Cl_4_ SC exhibits lower microstrain, lower defect density, higher *E*_*a*_ and higher resistivity. As a consequence, the fabricated Pb_2_CuGly_2_Cl_4_ SC X-ray detector enables an excellent operating stability with a low dark current drift of 1.20 × 10^–9^ nA mm^−1^ s^−1^ V^−1^ under a high electric field (120 V mm^−1^) and continuous X-ray irradiation. Besides, Pb_2_CuGly_2_Cl_4_ SC detector also endows a high sensitivity of 9250 μC Gy^−1^ cm^−2^. Furthermore, the Pb_2_CuGly_2_Cl_4_ nanocrystals are dispersed in water, and the obtained Pb_2_CuGly_2_Cl_4_ paste is blade-coated on TFT array to fabricate an X-ray imaging detector with a spatial resolution of 2.2 lp mm^−1^. The amino acids ligands metal cross-linked strategy broadens the types of 3D perovskites with suppressed ion migration, and the facile preparation technique of integrated X-ray imaging detectors presents a promising prospect for practical applications in the future.

## Supplementary Information

Below is the link to the electronic supplementary material.

Supplementary file1 (DOCX 13837 KB)
