# Supplementary material for: Face-/Edge-Shared 3D Perovskitoid Single Crystals with Suppressed Ion Migration for Stable X-Ray Detector
Source: Nanomicro Lett. 2025 Jun 23;17:310. doi: 10.1007/s40820-025-01788-z (PMC12185819; doi:10.1007/s40820-025-01788-z)
Supplement: Supplementary file 1 — Supplementary file1 (DOCX 13837 KB) [file 40820_2025_1788_MOESM1_ESM.docx]

Supporting Information for

**Face/Edge-Shared 3D Perovskitoid Single Crystals with Suppressed Ion Migration for Stable X-Ray Detector**

Zimin Zhang^1^*^#^*, Xiaoli Wang^2,3^*^#^*, Huayang Li^1*^, Dong Li^2^, Yang Zhang^2^, Nan Shen^1^, Xue-Feng Yu^2,3,4^, Yucheng Liu^5^, Shengzhong (Frank) Liu^5,6^, Haomin Song^7^, Yanliang Liu^2,3,4*^, Xingzhu Wang^8*^, Shi Chen^1*^

^1^Henan Key Laboratory of Quantum Materials and Quantum Energy, School of Future Technology, Henan University, Zhengzhou 450046, P. R. China

^2^Shenzhen Institute of Advanced Technology, Chinese Academy of Sciences, Shenzhen, Guangdong 518055, P. R. China

^3^University of Chinese Academy of Sciences, Beijing 100049, P. R. China

^4^Key Laboratory of Biomedical Imaging Science and System, Chinese Academy of Sciences, Shenzhen, Guangdong 518055, P. R. China

^5^Key Laboratory of Applied Surface and Colloid Chemistry, National Ministry of Education, Shaanxi Engineering Lab for Advanced Energy Technology, School of Materials Science and Engineering, Shaanxi Normal University, Xi’an 710119, P. R. China

^6^State Key Laboratory of Catalysis, Dalian National Laboratory for Clean Energy, Dalian Institute of Chemical Physics, Chinese Academy of Sciences, Dalian 116023, P. R. China

^7^Institute of Applied Physics and Materials Engineering, University of Macau, Taipa, Macau SAR 999078, P. R. China

^8^School of Electrical Engineering, University of South China, Hengyang, 421001, P. R. China

*^#^*Zimin Zhang and Xiaoli Wangcontributed equally to this work

*Corresponding authors. E-mail: [lhychem@henu.edu.cn](mailto:lhychem@henu.edu.cn) (Huayang Li); [yl.liu4@siat.ac.cn](mailto:yl.liu4@siat.ac.cn) (Yanliang Liu); [wangxz@sustech.edu.cn](mailto:wangxz@sustech.edu.cn) (Xingzhu Wang); [chenshi@henu.edu.cn](mailto:chenshi@henu.edu.cn) (Shi Chen)

**S1 Experimental Section and Calculation**

***DFT calculation:*** We used the DFT as implemented in the Vienna Ab initio simulation package (VASP) in all calculations. The exchange-correlation potential is described by using the generalized gradient approximation of Perdew-Burke-Ernzerhof (GGA-PBE). The projector augmented-wave (PAW) method is employed to treat interactions between ion cores and valence electrons. The plane-wave cutoff energy was fixed to 450 eV. Given structural models were relaxed until the Hellmann–Feynman forces smaller than -0.03 eV/Å and the change in energy smaller than 10^-4^ eV was attained. Grimme’s DFT-D3 methodology was used to describe the dispersion interactions among all the atoms in adsorption models. The Gamma-centered k-points samplings were set to 2 × 2 × 1 for modle.

***The space-charge-limited currents (SCLC) measurements:*** The trap density of the Pb_2_CuGly_2_X_4_ (X=Cl, Br) SCs can be calculated according to the following equation:

$n_{trap}=\frac{2V_{TFL}\varepsilon\varepsilon_{0}}{ⅇL^{2}}$

where the *V_TFL_*, *ε_0_*, and *L* denote the threshold voltage, vacuum permittivity, and the Pb_2_CuGly_2_X_4_ (X=Cl, Br) SC thickness, respectively.

***The measurement of sensitivity:*** The sensitivity (*S*) is defined as the response current density per unit dose rate:

$$S=\frac{I_{ph}-I_{dark}}{AD}$$

Where *I_ph_* is the photocurrent of device, *I_dark_* is the dark current, and $A$ is the device area, and *D* is the dose rate.

***The measurement of the detection efficiency:*** The theoretical efficiency of both devices can be estimated by the following equation [S1]:

*S_0_* = $\frac{(\frac{\varphi}{X})Ē\beta}{W_{\pm}} e\eta$

Where the *φ/X* denotes the number of photons per unit of exposure, *Ē* is the mean energy of the X-ray photons, the *W_±_* represents the ionization energy to create an electron-hole pair, *β*, *e* and *η* are the energy absorption efficiency of X-ray, elemental electron charge and charge collection efficiency, respectively. The *φ/X* is about 277068 photons mm^-2^ mR^-1^, which is equal to 3.16×10^12^ photons cm^-2^ Gy^-1^ (1 mR = 8.76×10^-6^ Gy) [S2]. The *Ē* for the 50 kV tube voltage is calculated to be about 42 keV according to the equation:

Ē = $\sum_{i} \rho\left( E_{i} \right)\triangle E_{i}$

where *ρ (E_i_)* and *△E_i_* denote the distribution probability and the energy bin width of X-rays, respectively. According to the UV-Vis-NIR absorbance spectrum, the *W_±_* of the Pb_2_CuGly_2_X_4_ (X=Cl, Br) SC are calculated to be 3.63 and 3.61 eV based on the empirical model:

*W_±_* = 2*E_g_* + 1.43 eV

The *β* of Pb_2_CuGly_2_X_4_ (X=Cl, Br) SC for 50 keV X-ray is calculated to be about 83% and 82%, respectively by the equation:

*β* =1-*e^(-ax)^*

where *a* is linear absorption coefficient of Pb_2_CuGly_2_X_4_ (X=Cl, Br) SC, and the *x* is corresponding SC thickness. As for the Pb_2_CuGly_2_X_4_ (X=Cl, Br) SC detector, the thickness of the Pb_2_CuGly_2_X_4_ (X=Cl, Br) SC are 1 mm and 0.8 mm, respectively. The *a* values of Pb_2_CuGly_2_X_4_ (X=Cl, Br) SC is 17.62 cm^-1^ and 21.81 cm^-1^ for 50 keV X-ray. The *η* is assumed to be 100%.

***The measurements of signal-to-noise ratio (***$\boldsymbol{SNR}$***):*** The signal-to-noise ($SNR$) values were calculated using the following equation:

$$SNR=\frac{I_{signal}}{I_{noise}}$$

Where $I_{signal}$​ is the detector signal current, obtained by subtracting the average dark current ($\bar{I}_{dark}$​) from the average X-ray response current ($\bar{I}_{ph}$​), and $I_{noise}$​ is the effective detector noise current, obtained by calculating the standard deviation of the response current:

$$I_{noise}=\sqrt{\frac{\sum_{i=1}^{N} \left( I_{i}-\bar{I}_{ph} \right)^{2}}{N}}$$

***The dark current (photocurrent) drift:*** The dark current (photocurrent) drift ($I_{drift}$) can be estimated by the following equation:

$$I_{drift}=\frac{I_{finish}-I_{begin}}{t\cdot s\cdot E}$$

Where $I_{finish}$represents the final current, $I_{begin}$ is the initial current at the beginning of the test, *t* represents the whole test time, *s* denotes the device area, and *E* is the electric field.

**S2 Supplementary Figures and Tables**

**Table S1** Crystallographic information of Pb_2_CuGly_2_X_4_ (X=Cl, Br)

**
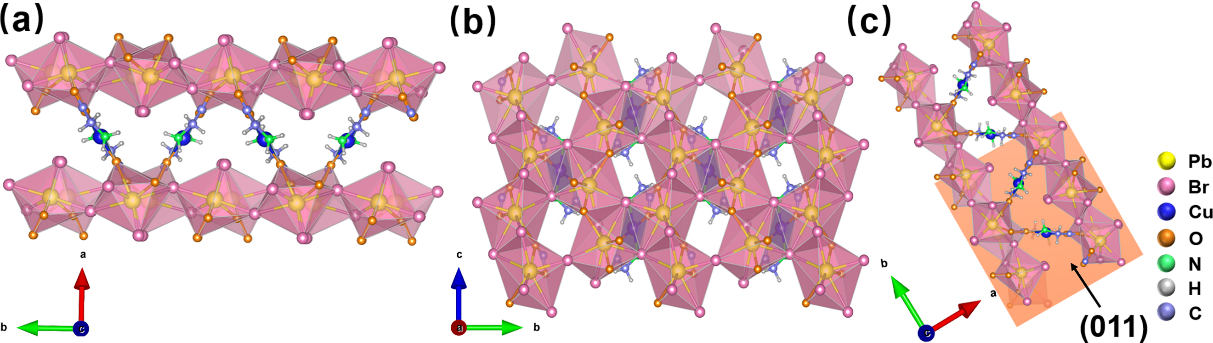
**

**Fig. S1** The crystal structure of Pb_2_Cu(O_2_C-CH_2_-NH_2_)_2_Cl_4_ observed along c axis (**a**) and a axis (**b**), and the (011) crystal plane (**c**)

**
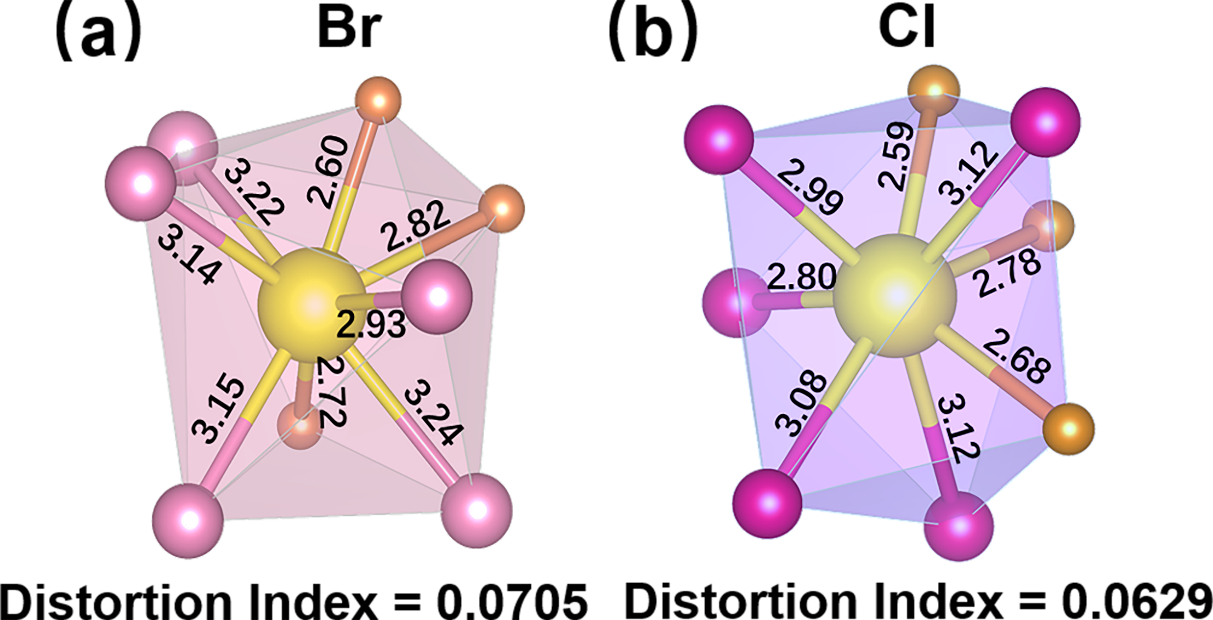
**

**Fig. S2** The structure of the [PbX_5_O_3_]^9-^ (X=Br, Cl) dodecahedral and the length of Pb-O and Pb-X

**Fig. S3** The Williamson–Hall (WH) plots of Pb_2_CuGly_2_X_4_ (X=Cl, Br) SC according to the XRD patterns

The corresponding micro strain calculated from Williamson–Hall (WH) plots was depicted in Figure S3. According to previous studies, microstrain will induce the formation of defects and hamper the stability of device. It can be seen that the derived microstrain of Pb_2_CuGly_2_Cl_4_ is 0.02, smaller than that of Pb_2_CuGly_2_Br_4_ (0.07). This result suggests a lower defect density and higher stability of Pb_2_CuGly_2_Cl_4_ SC.


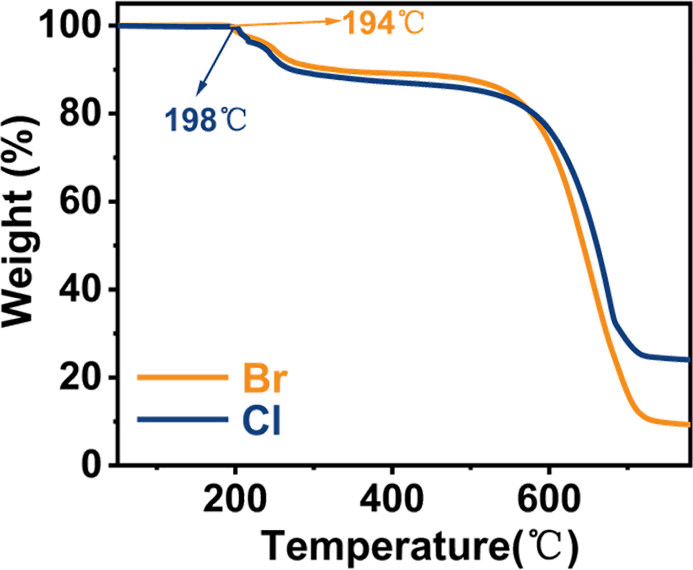


**Fig. S4** TGA curves of Pb_2_CuGly_2_X_4_ (X=Cl, Br) powder

**Table S2** The radius of the different ions

| Ions | Pb^2+^ | Br^-^ | Cl^-^ |
| --- | --- | --- | --- |
| Radius/nm | 0.119 | 0.195 | 0.181 |

**Table S2** lists the radii of halide ions and Pb^2+^ commonly used in perovskites to compare the electrostatic interaction forces between the X^-^ and the Pb^2+^, which can be calculated according to Coulomb's law:

$$F=\frac{kq_{1}q_{2}}{r^{2}}$$

where *F* is the interaction force between the two ions, $q_{1}$ and $q_{2}$ are the charge on the two point particles, *r* is the distance between the two particles, and *k* is the Coulomb's constant, which is about 9.0 ×10^9^ N m^2^ C^-2^ in air.

**
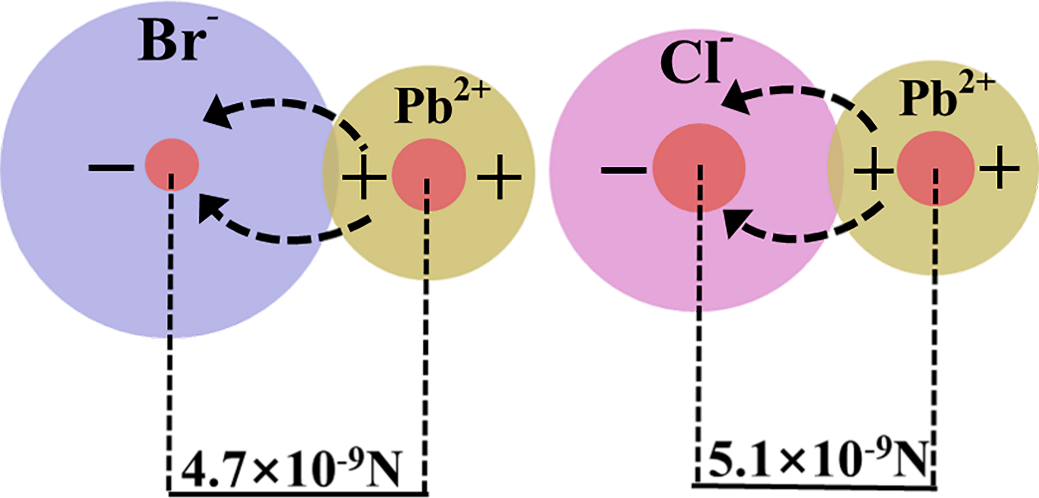
**

**Fig. S5** The schematic diagram of the electrostatic interaction force between Pb^2+^ and X^-^ (X=Br, Cl)

**Fig. S6** (**a**) The photographs of Pb_2_CuGly_2_X_4_ (X=Cl, Br) single crystal under light (left) and UV-light (310 nm). (**b**) The PL spectra of Pb_2_CuGly_2_X_4_ (X=Cl, Br) single crystal under excitation of 310 nm UV-light

**
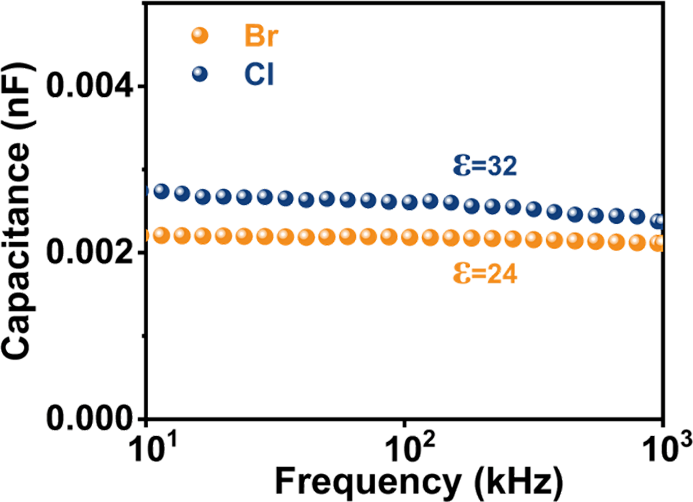
**

**Fig. S7** Frequency-dependent capacitance curves of Pb_2_CuGly_2_X_4_ (X=Cl, Br) SC

The capacitance-frequency curves of Pb_2_CuGly_2_X_4_ (X=Cl, Br) were measured in the dark over a wide frequency range from 10 to 1000 kHz via a TZDM-RT-1000 dielectric temperature spectrometer. The relative permittivity ($\varepsilon$) was calculated according to the following Eq:

$$\varepsilon=\frac{cd}{\varepsilon_{0}A}$$

where $A$ and $d$ are the device area and the thickness of SC, respectively, $\varepsilon_{0}$ is

the vacuum permittivity, c is the capacitance. The $\varepsilon$ of the Pb_2_CuGly_2_X_4_ (X=Cl, Br) SC were calculated to be 32 and 24, respectively.


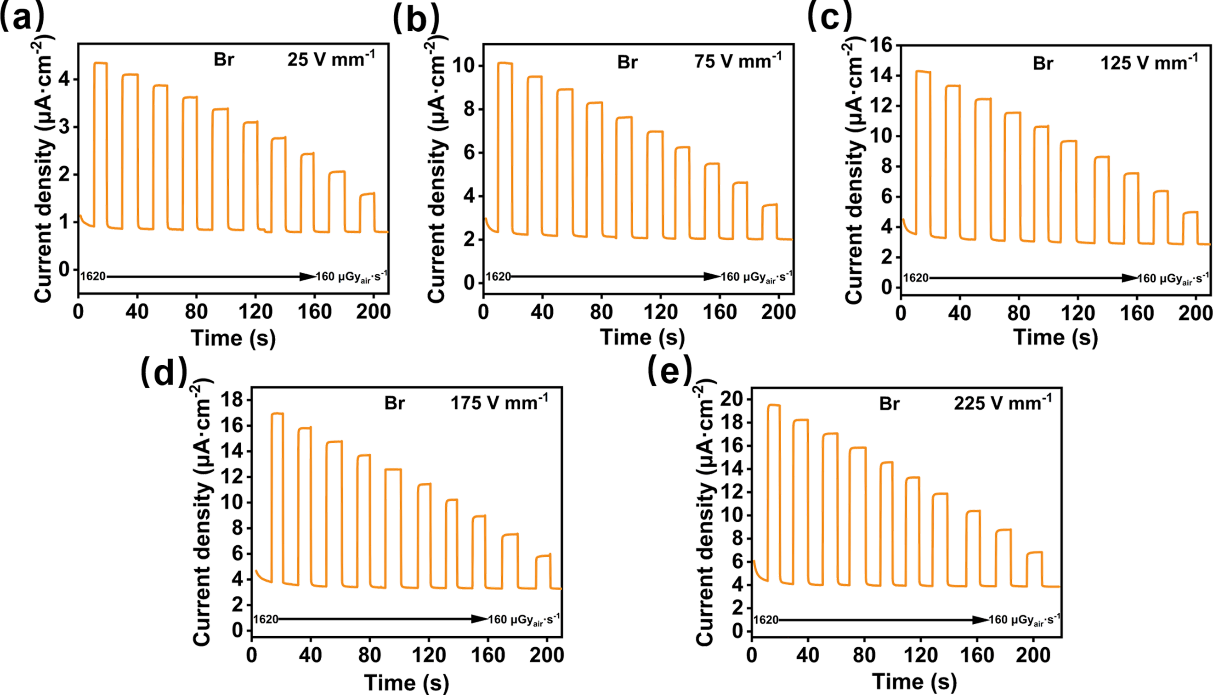


**Fig. S8** The time-resolved current density of Pb_2_CuGly_2_Br_4_ SC detector varies with the dose rates under different electric field

**
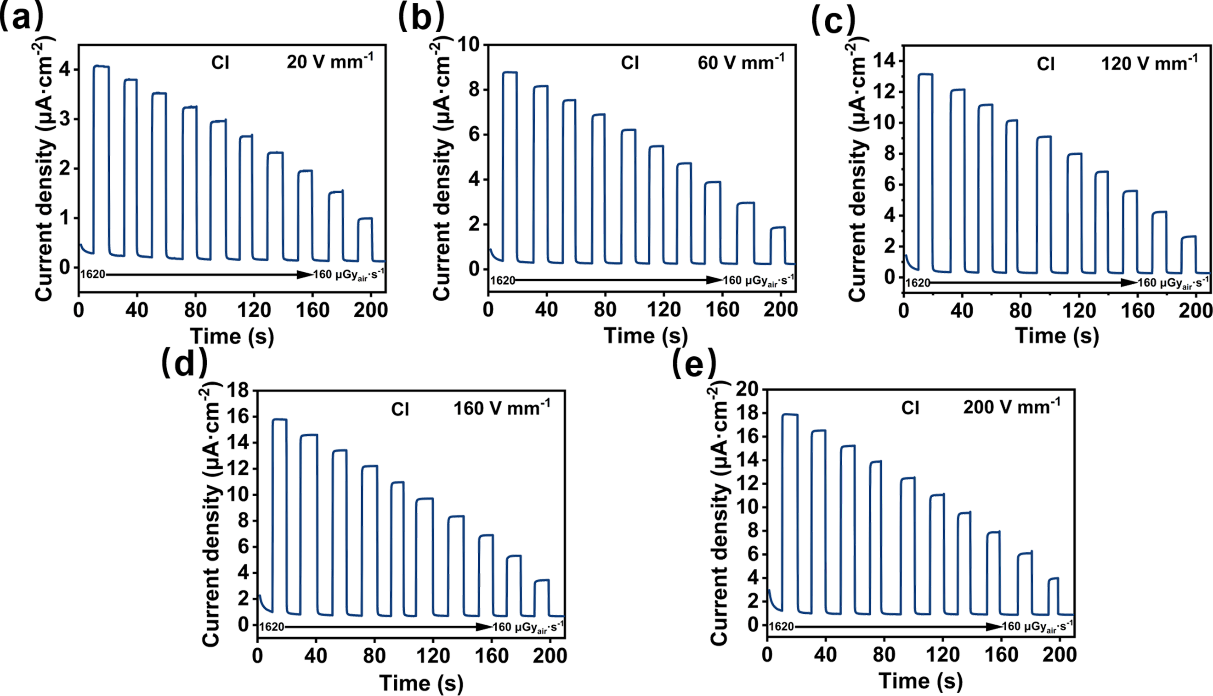
**

**Fig. S9** The time-resolved current density of Pb_2_CuGly_2_Cl_4_ SC detector varies with the dose rates under different electric field


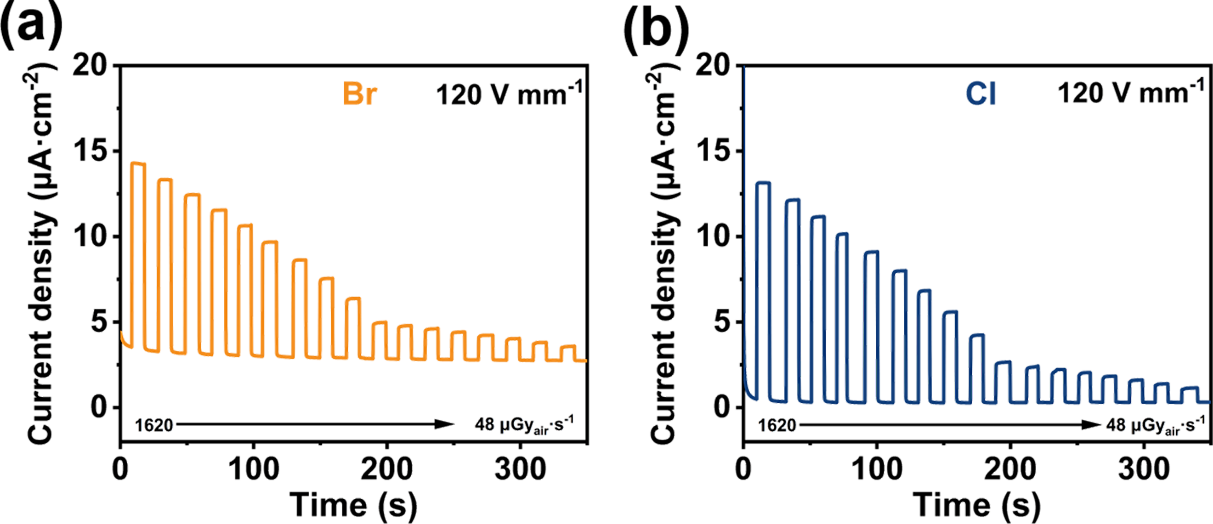


**Fig. S10** The time-resolved current density of Pb_2_CuGly_2_Br_4_ (a) and Pb_2_CuGly_2_Cl_4_ (b) SC detectors varies with the dose rates at the electric field of 120 V mm^-1^

**
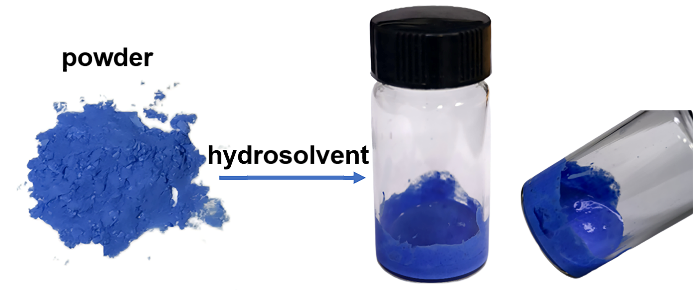
**

**Fig. S11** The photograph of the Pb_2_CuGly_2_Cl_4_ powder and paste


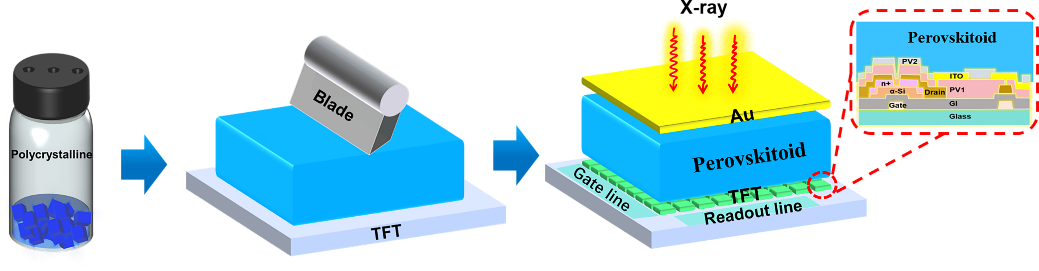


**Fig. S12** The preparation process of Pb_2_CuGly_2_Cl_4_ TFT array detector and inset shows the enlarged internal structure of TFT substrate


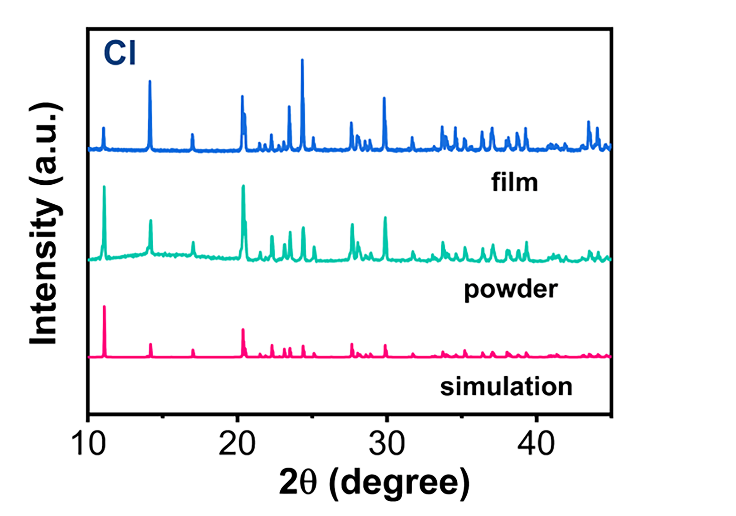


**Fig. S13** The XRD patterns of Pb_2_CuGly_2_Cl_4_ film

**Table S3** The comparison of X-ray detection performance based on different semiconductors

| Device structure | X-ray source [keV] | Electric field [V mm^-1^] | Sensitivity  [μC·Gy_air_^-1^·cm^-2^] | Detection limit [nGy_air_·s^-1^] | Dark drift  [nA∙cm^-1^∙s^-1^∙V^-1^] | Refs. |  |
| --- | --- | --- | --- | --- | --- | --- | --- |
| α-Se | 20 | 10000 | 20 | 5.5×10^3^ |  | [S3] |  |
| CdZnTe | 80 | 250 | 2400 | 5×10^4^ |  | [S4] |  |
| Pt/MAPbBr_3_/Pt | 100 | 10 | 2.45×10^4^ | 54 | 3.88×10^-5^ | [S5] |  |
| Au/MA_0.42_FA_0.58_PbI_3_/ATPES/FTO | 40 | 71.4 | 1.16×10^6^ | 37.4 | 0.25 (-5V) | [S6] |  |
| FTO/CsPbBr_3_/Au | 50 | 100 | 9085 | 103.6 | 7.1×10^-4^ | [S7] |  |
| EGaIn/CsPbBr_3_/Au | 120 | 500 | 4.6×10^4^ | 10.81 | 1.68×10^-6^ | [S8] |  |
| Au/CsPb_2_Br_5_/Au | 40 | 625 | 8865.6 | 12.7 |  | [S9] |  |
| Au/CsPbBr_2.9_I_0.1_/Au | 70 | 100 | 2.75×10^5^ | 18 |  | [S10] |  |
| Au/α-FAPbBr_3_/Au | 70 | 5 | 1.67×10^5^ | 1.1 |  | [S11] |  |
| Ag/(4ABA)PbI_4_/Ag | 50 | 5 | 572 | 7.50 | 5.18×10^-8^ | [S12] |  |
| Au/(F-PEA)_2_PbI_4_/C_60_/BCP/Cr | 120 | 133 | 3402 | 23 | 4.9×10^-8^ | [S13] |  |
| Au/(DGA)PbI_4_/Au | 40 | 1200 | 4869 | 95.4 | 5.97×10^-7^ | [S14] |  |
| Au/(4AEPy)PbI_4_/C_60_/BCP/Cr | 120 | 200 | 5627 | 20 | 1.9×10^-8^ | [S15] |  |
| Au/BDAPbI_4_/Au | 40 | 310 | 242 | 430 | 6.06×10^-9^ | [S16] |  |
| Ag/BA_2_PbBr_4_/Ag | 50 | 920 | 726 | 8.2 |  | [S17] |  |
| Cu/Pb_2_CuGly_2_Cl_4_/Cu | 50 | 120 | 7020 | 912.6 | 1.20×10^-8^ | This work |  |

**Fig. S14** Microphotograph of the different region of TFT array

**
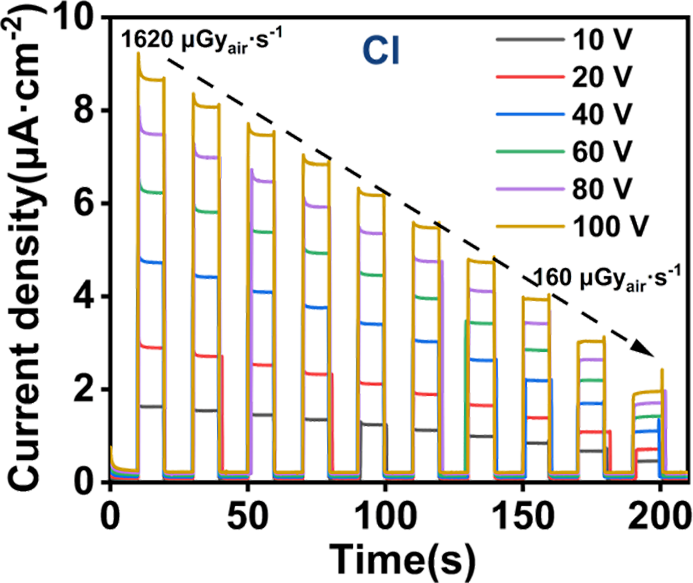
**

**Fig. S15** The time-resolved current density of Pb_2_CuGly_2_Cl_4_ TFT array detector varies with the dose rates under different electric field

**Supplementary References**

[S1] S. Tie, W. Zhao, D. Xin, M. Zhang, J. Long, et al., Robust fabrication of hybrid lead-free perovskite pellets for stable x-ray detectors with low detection limit. Adv. Mater. **32**(31), 2001981 (2020). <https://doi.org/10.1002/adma.202001981>

[S2] J. M. Boone, J. A. Seibert, An accurate method for computer-generating tungsten anode x-ray spectra from 30 to 140 kv. Med Phys. **24**(11), 1661-1670 (1997). <https://doi.org/10.1118/1.597953>

[S3] S. O. Kasap, X-ray sensitivity of photoconductors: Application to stabilized a-se. J Phy D. **33**(21), 2853-2865, (2000). <https://doi.org/10.1088/0022-3727/33/21/326>

[S4] S. Tokuda, H. Kishihara, S. Adachi, T. Sato, Preparation and characterization of polycrystalline cdznte films for large-area, high-sensitivity x-ray detectors. J Mater Sci. **15**(1), 1-8 (2004). <https://doi.org/10.1023/A:1026297416093>

[S5] D. Liu, Y. Zheng, X. Y. Sui, X. F. Wu, C. Zou, et al., Universal growth of perovskite thin monocrystals from high solute flux for sensitive self-driven x-ray detection. Nat. Commun. **15**(1), 2390 (2024). <https://doi.org/10.1038/s41467-024-46712-y>

[S6] W.-G. Li, X.-D. Wang, Y.-H. Huang, D.-B. Kuang, Ultrasound-assisted crystallization enables large-area perovskite quasi-monocrystalline film for high-sensitive x-ray detection and imaging. Adv. Mater. **35**(31), 2210878 (2023). <https://doi.org/10.1002/adma.202210878>

[S7] X. Du, Y. Liu, W. Pan, J. Pang, J. Zhu, et al., Chemical potential diagram guided rational tuning of electrical properties: A case study of cspbbr3 for x-ray detection. Adv. Mater. **34**(17), 2110252 (2022). <https://doi.org/10.1002/adma.202110252>

[S8] Y. Hua, G. Zhang, X. Sun, P. Zhang, Y. Hao et al., Suppressed ion migration for high-performance x-ray detectors based on atmosphere-controlled efg-grown perovskite cspbbr3 single crystals. Nat. Photonics **18**(8), 870-877 (2024). <https://doi.org/10.1038/s41566-024-01480-5>

[S9] X. Feng, L. Zhang, B. Zhang, J. You, K. Li et al., Ligand-assisted growth of 2d perovskite single crystal for highly sensitive x-ray detectors. Adv. Funct.Mater. **34**(37), 2402166 (2024). <https://doi.org/10.1002/adfm.202402166>

[S10] R. Shi, J. Pi, D. Chu, B. Jia, Z. Zhao et al., Promoting band splitting through symmetry breaking in inorganic halide perovskite single crystals for high-sensitivity x-ray detection. ACS Energy Lett. **8**(11), 4836-4847 (2023). <https://doi.org/10.1021/acsenergylett.3c01661>

[S11] D. Chu, B. Jia, N. Liu, Y. Zhang, X. Li et al., Lattice engineering for stabilized black fapbi3 perovskite single crystals for high-resolution x-ray imaging at the lowest dose. Sci. Adv. **9**(35), eadh2255 <https://doi.org/10.1126/sciadv.adh2255>

[S12] Q. Fan, Y. Ma, S. You, H. Xu, W. Guo et al., Dion–jacobson phase perovskite crystal assembled by π-conjugated aromatic spacer for x-ray detectors with an ultralow detection limit. Adv. Funct. Mater. **34**(9), 2312395 (2024). <https://doi.org/10.1002/adfm.202312395>

[S13] H. Li, J. Song, W. Pan, D. Xu, W.-a. Zhu et al., Sensitive and stable 2d perovskite single-crystal x-ray detectors enabled by a supramolecular anchor. Adv. Mater. **32**(40), 2003790 (2020). <https://doi.org/10.1002/adma.202003790>

[S14] B. Zhang, T. Zheng, J. You, C. Ma, Y. Liu et al., Electron-phonon coupling suppression by enhanced lattice rigidity in 2d perovskite single crystals for high-performance x-ray detection. Adv. Mater. **35**(7), 2208875 (2023). <https://doi.org/10.1002/adma.202208875>

[S15] W. Li, X. Feng, K. Guo, W. Pan, M. Li et al., Prominent free charges tunneling through organic interlayer of 2d perovskites. Adv. Mater. **35**(18), 2211808 (2023). <https://doi.org/10.1002/adma.202211808>

[S16] Y. Shen, Y. Liu, H. Ye, Y. Zheng, Q. Wei et al., Centimeter-sized single crystal of two-dimensional halide perovskites incorporating straight-chain symmetric diammonium ion for x-ray detection. Angew Chem Int Ed. **59**(35), 14896-14902 (2020). <https://doi.org/10.1002/anie.202004160>

[S17] X. Xu, Y. Wu, Y. Zhang, X. Li, F. Wang et al., Two-dimensional perovskite single crystals for high-performance x-ray imaging and exploring mev x-ray detection. Energy Environ Mater. **7**(1), e12487 (2024). <https://doi.org/10.1002/eem2.12487>
